# Progress in Renal Diseases

**Published:** 1902-07-12

**Authors:** 


### Progress in Renal Diseases.

**Urology.**—Cavazza[1] finds that urobilin is greatly increased at the commencement of severe chlorosis and in relapses. It is well known that an increase follows any great blood destruction as in malaria or hæmoglobinuria. In chlorotics, too, over-exertion, fever, or cold bathing will bring on an increase, though in normal health there is no such result. Hence he thinks that the red cells are in a feeble state in this disease. The toxicity of urine in pregnancy has been carefully investigated by R. W. Stewart,[2] who concludes that the toxicity is generally the result of infection by micro-organisms introduced by the catheter, though the elimination of poisonous matters may in some cases lead to a direct infection. Albumen has been found by H. Cramer[3] in the form of an emulsion, especially during coma and uræmia, but nothing is known of the cause of this condition, though the prognosis seems to be very bad. *Bacteriuria* in zymotic diseases has been more commonly recognised of late. Thus C. T. Lewis[4] shows that in plague the special bacillus may occur and remain long during convalescence in large numbers. In typhoid the bacillus is most often seen in severe cases and at a late stage. Pyuria is then often present too. He thinks that the common estimate of the frequency of bacilli in typhoid urine—viz. 25 per cent. of all cases—is too high, and that in severe cases they occur at the height of the disorder, with albuminuria, and soon disappear unless cystitis has been set up. There is, however, a bacilluria of convalescence which lasts long and may follow any type of the fever without any special symptoms. In scarlatina he found streptococci in seven cases out of 51. Some of these resembled in their reactions the streptococcus of Baginsky, which he found constantly in the blood of scarlatinal patients. In diphtheria the bacillus has been found by various writers, but Lewis failed to isolate it in 43 examinations from 17 cases. J. O. Symes[5] thinks that in typhoid the bacilli are not present in more than 10 per cent. of the cases, and in chronic phthisis the tuberculosis bacillus occurs in 50 per cent. In pyelonephritis he found staphylococci, streptococci, and diplococci. Alkaline cystitis is nearly always due to staphylococci, but other organisms, such as proteus, B. coli, and streptococci, may be present. The antiseptic power of urotropin is most marked in acid urine at the body temperature. It is probably due to some compound which it forms, and not to the liberation of formaldehyde. Its effect upon B. typhosus and lactis aerogenes is marked, while that on other organisms is less reliable. In pyelonephritis the effects are poor, probably because the organisms are deeply embedded and the urine is often orginally alkaline, while, on the other hand, in cystitis due to prostatic troubles capital results are obtained, for the urine here is acid on leaving the kidney. Cystitis due to B. coli or to the gonococci, though relieved, is rarely cured by the drug. In any case, owing to its rapid excretion it is desirable to give a dose the last thing at night.

**Cyclic albuminuria.**—W. E. Huger[6] gives a useful summary of the different theories which have been held as to this disorder. Some like Osler insist that it is due to a pathological change; some think it due to the presence of oxalates or to changes in the vasomotor mechanism which affects the glomeruli only; others again think that albumen can occur physiologically, but no post-mortem has been made on anyone suffering from cyclic albuminuria, and therefore the real pathology is unknown. The writer does not hold that importance should be attached to the points usually laid stress on—viz. the duration of the affection, the number of hours during which albumen is daily excreted, its amount, or the presence of casts. Taking all cases together, we may say that a few develope true Bright's disease, while the vast majority get quite well. Prolonged rest in bed is the only thing which has any effect on the condition.

**Urinary Pigments.**—Indigo has been observed by

McPhedrun and Goldie [7] in a great smoker who led a sedentary life. His urine at other times contained much indican, but no cause for the presence of indigo itself could be found. The most frequent cause of green or blue urine seems to be the methylene blue used in colouring sweets. Decolorisation can be effected by adding a cold solution of caustic potash, and the colour may often be increased by boiling with a little acetic acid. Parkes Weber thinks that other aniline dyes may be excreted in the urine, as methylene blue. Schölberg describes a new purple pigment, probably congenital, and allied to hæmato-porphyrin. For the detection of indican A. Klett [8] recommends the addition to the urine of half its volume of HCl. of 25 per cent. strength, and a few drops of ammonium persulphate. Chloroform is coloured blue if shaken up with this mixture when indican is present. *As a test for albumen* F. C. Fuhs [9] recommends equal parts of carbolic acid and glycerine. This solution is added to an equal bulk of the urine to be tested, and shaken up. If a clear transparent liquid results there is no albumen present, or at least less than 0·1 per cent. If there is albumen a white turbidity appears which does not precipitate on standing. The test seems to compare well with the ordinary ones, and saves carrying about the troublesome nitric acid, or boiling.

**Surgical Treatment.**—Edebohls [10] records a remarkable and almost incredible series of 18 cases of chronic Bright's disease in which he has operated without a death, with the result that the symptoms either disappeared altogether or were greatly improved. He was led to undertake this plan of treatment in consequence of the cure of the chronic nephritis in some five patients on whom he had operated for floating kidney. In the course of such a nephropexy he exposes and lifts out the kidneys, and carefully strips off the capsule, which he cuts away. The organ thus bared is anchored in the surrounding fat, and not only are fibrous adhesions formed, but a number of large vessels are formed and enter the kidney. It is this new blood supply which, he thinks, is the means by which the healthy action of the kidney is restored. In the course of these operations he noticed a curious fact, that in nearly half the cases with symptoms of chronic nephritis one kidney was apparently healthy. This, of course, is opposed to what is usually found in post-mortems upon patients dying from the disease; and he is therefore led to the belief that the disease is at first often unilateral, and that in time both kidneys gradually become involved. The existence of nephritis in his patients had been known before operation for a period ranging up to six years. In nine patients operated upon more than a year ago eight show normal urine. Harrison, in 1896, advocated incision into the kidney in acute cases where there was evidence of increased tension, and several cases where benefit resulted from this procedure have been recorded. Israel has confirmed this view, and Poussen showed that temporary improvement followed incision in a case of interstitial nephritis.[11] F. C. Boyd concludes that incision is justifiable in acute nephritis where there is anuria which will not yield to other methods. Edebohls' decapsulation in chronic disease opens up a still wider field and merits careful consideration.

**Floating Kidney** is treated by J. Ross Watts [12] by the application of wing-shaped pieces of sheet lead to the abdomen, nearly meeting in the middle line, and stitched to long, well-fitting corsets. The lead should weigh 5 lb. to the square foot, and a pattern should be first cut out on the body in brown paper, not reaching any bony prominence by half an inch. The leads are cut out from these patterns, sewn inside Holland covers with broad edges, applied to the patient while lying down, and moulded by hand. The corset is then put over them and laced up, and the leads fixed to it temporarily while the patient is still lying down. They are then sewn on exactly in the same part of the corset. This leaded corset is to be put on daily while the patient is lying down, after the kidney has been pushed back to its normal position, and it should only be removed after she has lain down. After three or four months the steady pressure thus exerted causes the organs to become fixed in their right position in the majority of cases. Eighteen instances are given of the use of this method. Straight-fronted corsets are advisable. Mansell Moullin [13] notices two types of movable kidney. The first not only moves down bodily with the diaphragm but fails to ascend with it, while the second moves normally as a whole but the upper end drops forward almost to a horizontal position and is pressed against the pylorus or bowel. This is harder to recognise, but may often cause symptoms of gastric ulcer and dyspepsia in middle-aged women. Again, it may cause symptoms like those of mild biliary colic and even jaundice, but the pain will sometimes cease if the patient lies down for a few hours. He mentions two of these cases cured by nephropexy. The cause of the much greater frequency of movable kidney on the right side than on the left is the shallowness of the right renal recess from the pushing forward of the transverse lumbar processes by the rotation of those vertebræ to form the left lumbar curve which is present in all right-handed people. He agrees with Edebohls that appendicular trouble is often associated with right movable kidney. The affection of the appendix may be of a slight character, but usually when the patients come to us it has gone on so far that it is necessary to remove the appendix as well as to stitch the kidney. *Lactescence of the blood serum* is generally present when there is a kidney lesion, and is due to the presence of small highly refractive granules formed of fatty bodies. Widal and Papillon [14] regard them as due to the fatty degeneration of leucocytes. It is, however, rare in interstitial nephritis, almost constant in tubular nephritis, and often follows an acute infective disease. Debove thinks that chronic nephritis may arise from this blood state. What are the exceptions to the rule that *hypertrophy of the heart* follows interstitial nephritis? The recognised cases, says F. O. Boyd,[15] are senility, tuberculosis, and cachexias; but he quotes also instances showing that chlorotics with small aortas and aplasia of the vessels should be added to the list, since in them the heart also fails to respond to the condition. It is to be noted that the nephritis is rapidly fatal in these latter cases, probably because in the absence of hypertrophy there is nothing to increase the excretion of effete urinary matters. Mayet [16] has found marked hypertrophy of the ventricle in rabbits in which a toxic nephritis had been produced by injections of cantharidine. These had been given in the course of experiments for the transplantation of cancer. The hypertrophy took place within a few months. Carter[17] records the results of 500 measurements of arterial pressure by the sphygmometer, and remarks that the average normal mean arterial pressure is 113–116 mm. In acute nephritis the pressure varies with the amount of albumen, but when following an acute infectious disease there is little rise. In chronic nephritis it is much higher. The best drug for lowering pressure is sodium nitrite, two or three grains being given every two or four hours. It lowers the pressure about 12 mm. and the effects last an hour and a-half. If uræmia is also present, bleeding and saline injections should also be employed. Improvement almost invariably follows. Pressure is low in all forms of anæmia and in pure aortic regurgitation. Intense headache is sometimes met with in uræmia with high pressures. Scherb[18] refers to Marie's success in relieving this pain by lumbar puncture in six cases, and adds an instance from his own practice. On two occasions lumbar puncture was performed, and 20 or 30 c.cm. of fluid were drawn off, with striking relief after a few hours. He thinks it diminishes the cerebral toxæmia and relieves the intracranial pressure. Besides slight cerebral disturbances, such as giddiness, uræmia may produce more or less prolonged mental disorders. Thus Carriès and Cullere[19] give instances of insanity with hallucinations, delusions, and maniacal excitement, followed by melancholia, produced, as they believe, by uræmia. Venesection and saline transfusion seem to be indicated in such cases.

1 Treatment, February. 2 Ibid. 3 Med. Rec., February 15. 4 Med. Chron., January. 5 Bristol Med. Chir. Jour., March. 6 Johns Hopkins Bulletin, April. 7 Treatment, February. 8 Lancet, February 1. 9 Med. Rec., p. 374. 10 Med. Rec., December 21. 11 Scottish Med. and Surg. Jour., March. 12 Edin. Med. Jour., December. 13 Clin. Jour., March 12. 14 Scottish Med. and Surg. Jour., March. 15 Ibid. 16 Brit. Med. Jour., February 8. 17 Amer. Jour. Med. Sci., December. 18 Lancet, March 1. 19 Lancet, January 4.

